# Hepatic steatosis, carotid plaques and achieving MDA in psoriatic arthritis patients starting TNF-α blockers treatment: a prospective study

**DOI:** 10.1186/ar4049

**Published:** 2012-10-04

**Authors:** Matteo Nicola Dario Di Minno, Rosario Peluso, Salvatore Iervolino, Roberta Lupoli, Anna Russolillo, Giovanni Tarantino, Raffaele Scarpa

**Affiliations:** 1 Department of Clinical and Experimental Medicine, Reference Centre for Coagulation Disorders, Federico II University, Via Sergio Pansini 5, Naples, 80129 Italy; 2 Department of Clinical and Experimental Medicine, Rheumatology Research Unit, Psoriatic Arthritis Clinic, Federico II University, Via Sergio Pansini 5, Naples, 80129 Italy; 3 Rheumatology and Rehabilitation Research Unit, I.R.C.C.S. "Salvatore Maugeri" Via Bagni Vecchi 1, Telese Terme (BN), 82037 Italy

## Abstract

**Introduction:**

We prospectively evaluated whether hepatic steatosis (HS) and the presence of carotid plaques (CPs) impacts on achieving minimal disease activity (MDA) in psoriatic arthritis (PsA) patients starting tumor necrosis factor (TNF)-α blockers treatment.

**Methods:**

Before starting treatment with TNF-α blockers, consecutive PsA subjects with an active disease were evaluated for the presence of the metabolic syndrome (MetS), HS and CPs. The incidence of MDA was evaluated 12 and 24 months later.

**Results:**

Among 270 PsA subjects, 91 (33.7%) exhibited the MetS, 58 (21.5%) CPs and 76 (28.1%) HS. At the 12-month follow-up, 98 (36.3%) individuals achieved MDA. Compared with those who did, a higher prevalence of the MetS, HS and CPs was found in subjects who did not achieve the MDA (*P *always < 0.001). After adjusting for the MetS and for all the other demographic/clinical characteristics analyzed, the presence of HS and CPs at baseline independently predicted the risk of not achieving MDA (Hazard Ratio: 1.91, 95% confidence interval (CI): 1.04 to 3.38, *P *= 0.035 and Hazard Ratio: 3.21, 95%CI: 1.64 to 6.29, *P *= 0.001, respectively). Separate Kaplan-Meier survival models confirmed this (Log-Rank: 12.894, *P *< 0.001 and Log-Rank: 12.849, *P *< 0.001, respectively). Compared with those without, progressively increasing Hazard Ratios of not achieving MDA were found in those with HS, CPs or HS + CPs at baseline. Moreover, the presence of HS and/or CPs predicted the risk of relapse during the additional 12-month follow-up (Hazard Ratio: 2.85, 95%CI: 1.27 to 6.37, *P *= 0.011 and Hazard Ratio: 3.17, 95%CI: 1.57 to 6.41, *P *= 0.001 respectively).

**Conclusions:**

HS and/or CPs at baseline are negative predictors of achieving and maintaining MDA.

## Introduction

Psoriatic arthritis (PsA) is a chronic arthropathy associated with psoriasis, marked by an axial and/or peripheral joint involvement [1]. In addition to joint manifestations, subjects with PsA exhibit an abnormally high cardiovascular (CV) risk and, in turn, risk of the metabolic syndrome (MetS), one of its major vascular risk factors (VRFs) [2,3]. Accordingly, as compared with healthy controls, PsA patients exhibit a higher than normal platelet reactivity [4] and a higher than normal prevalence of hepatic steatosis (HS) [5] and of carotid plaques (CPs) [6]. All these conditions are well known markers of atherosclerosis and of CV risk [7,8].

Chronic inflammation may interact with VRFs, leading to a further increase of the CV risk in PsA patients [9,10]. In addition to being involved in the inflammatory process, most cytokines (TNF-α, IL-1, IL-6) play a role in the genesis and in the progression of atherosclerosis [11]. Both HS and CPs are strongly influenced by the severity of chronic inflammation [12-15] and are directly correlated with the MetS and its features.

Compared with those with minimal disease activity (MDA), PsA subjects with an active disease show an exaggerated worsening of HS degree [16] and an increased prevalence of CPs [17]. Thus, in addition to being associated with a cardio-metabolic impairment, CPs and/or HS may be markers of the severity of the inflammatory process in PsA subjects [12-15].

In view of this, we prospectively evaluated whether, regardless of the presence of MetS, the presence of HS and/or CPs at baseline impacts *per se *on the achieving of MDA in subjects with PsA who start a treatment with TNF-α blockers.

## Materials and methods

During a three-year period (January 2007 to January 2010), all patients with a diagnosis of PsA (according to the Classification Criteria for Psoriatic Arthritis (CASPAR) criteria) [1] who were non-responders to traditional disease modifying anti-rheumatic drugs (DMARDs) and who were referred to the Regional Reference Center for the treatment of spondyloarthropathies of the Federico II University of Naples to start treatment with TNF-α blockers, were evaluated for enrollment in this study. For all enrolled subjects, exclusion criteria were: lack of informed consent signature, age < 18 years, previous treatment with TNF-α blockers, adequate clinical response to traditional DMARDs treatment, malignancy, hematologic diseases, autoimmune diseases other than PsA, unstable medical conditions, ongoing pregnancy, history of venous and/or arterial thrombosis and of alcohol abuse. After approval of the study by the local Ethics Committee and after informed consent signature, data about age, gender, height, weight, disease duration, disease activity, previous and/or current treatments and vascular risk factors were collected from all patients as previously described [13]. All PsA subjects had clinically active disease at the time of enrollment and were classified into different clinical subsets (Moll and Wright criteria) [18]. Briefly, PsA patients were classified as having axial disease if they had at least grade 2 unilateral sacroiliitis in the presence of a combination of inflammatory back pain plus back stiffness. The PsA patients were classified as pure axial if they had no peripheral joint involvement, mixed if they had both peripheral joint arthritis and axial disease, or as having only peripheral joint involvement. The rare mutilans form was diagnosed in the presence of distal interphalangeal joint bone resorption (osteolysis), 'pencil-in-cup' radiographic findings or telescoping motion of the digit. According to the National Cholesterol Education Program (NCEP) criteria [19], abdominal obesity was defined as a waist circumference ≥102 cm for men and ≥88 cm for women; hypertriglyceridemia, as triglyceride levels ≥150 mg/dL; hypercholesterolemia with low-HDL cholesterol as a total cholesterol ≥200 mg/dL with HDL-cholesterol < 40 mg/dL for men and < 50 mg/dL for women; hypertension as a blood pressure ≥130 and/or 85 mmHg; and impaired fasting glucose (IFG) as a fasting glucose ≥100 mg/dL. Patients were defined as having the MetS if three or more of these VRFs were present. In addition, the presence of CPs and the presence/severity of HS were evaluated in each PsA subject according to previously validated scanning protocols [13,16]. All the carotid artery ultrasound examinations were performed by the same operator. After a five minute rest in the supine position, the subjects underwent a bilateral carotid ultrasonography (US) with a 7.5 to 12 MHz linear-array transducer and a duplex scanner (ESAOTE MyLab 25Gold, Genoa, Italy). The ultrasound evaluation was performed longitudinally and transversally by using gray-scale and color-Doppler US imaging. The scan protocol requires the full-length visualization of the near and far wall of the right and left common carotid artery (CCA) and of the bulb in three different projections (anterior, lateral and posterior). The intima-media thickness (IMT) was measured in each of the three projections in the CCA and bulb and the presence of CPs was defined for IMT ≥1.3 mm. The reproducibility of the vascular measurement was evaluated in 20 subjects within 1 week from the first ultrasonographic examination. The overall Pearson's r value for the IMT measurements was 0.89 (*P *< 0.001). The presence of HS was assessed with a US diagnostic system (Logiq P5, General Electric, Milan, Italy) with a 3.5-MHz convex probe. All the US determinations were made by the same trained operator. The intra-operator variability, as evaluated in 20 subjects within 1 week from the first ultrasonographic examination, showed an overall r value of 0.92 (*P *< 0.001). As previously validated [16], the presence/severity of HS was defined by comparing the echogenicity of the liver and of the kidney cortex: Grade 0 (absent): iso-echogenicity; Grade 1 (mild): diffuse and homogeneous hyper-echogenicity; Grade 2 (moderate): attenuation of the ultrasonic beam rear; Grade 3 (severe): lack of diaphragm profile visualization. The presence of CPs and of a moderate/severe (2^nd^/3^rd ^degree) HS were recorded in all subjects at baseline (T0). At T0 as well as every three months after having started the treatment with TNF-α blockers, all PsA subjects underwent a complete clinical rheumatologic and laboratory evaluation that included: tender joint count (TJC, *n *= 78), swollen joint count (SJC, *n *= 76), tender entheseal count (according to the Maastricht Ankylosing Spondylitis Enthesitis Score), psoriasis area severity index (PASI), health assessment questionnaire (HAQ), visual analog scale for pain (VAS), patient global disease activity VAS score, erythrocyte sedimentation rate (ESR) and C-reactive protein (CRP). The data obtained at 12 months were recorded as (T1). During the first 12 months of follow-up (from T0 to T1), PsA subjects were classified as having achieved MDA when fulfilling 5 or more of the following 7 outcome measures at T1: TJC ≤1; SJC ≤1; PASI ≤1 or body surface area ≤3; VAS for pain ≤15; patient global disease activity VAS score of ≤20; HAQ ≤0.5 and tender entheseal points ≤1 [20]. Otherwise, they were considered as not having achieved MDA (non-MDA). To test the achieving of sustained-MDA (s-MDA), those who had achieved MDA, were re-evaluated every 3 months thereafter during the additional 12-months (T2). s-MDA was defined as fulfilling the same criteria employed for MDA in all the visits carried out between T1 and T2.

The present study protocol has been approved by the Federico II University Local Ethic Committee.

### Statistical analysis

Statistical analysis was performed with the SPSS 16 system (SPSS Inc., Chicago, IL, USA). Continuous data were expressed as means ± SD; categorical variables were expressed as percent. The t-test was performed to compare continuous variables; the chi-square test was employed to analyze categorical data. When the minimum expected value was < 5, the Fisher's exact test was used.

A Kaplan-Meier survival model (with the Log-Rank test) was adopted to evaluate the cumulative achieving MDA according to the presence of HS or CPs. To adjust for all the other variables (with emphasis on the presence of the MetS) and to evaluate the achieving of MDA (or of s-MDA), separate COX regression analyses (stepwise method) were adopted, with the achieving MDA (or s-MDA) as the dependent variable and the MetS, CPs, HS, number of CV risk markers, obesity, hypertension, hypercholesterolemia, hypertriglyceridemia, impaired fasting glucose, smoking habit, TJC, SJC, tender entheseal count, PASI, HAQ, VAS for pain, patient global VAS, ESR, CRP, concomitant treatment with methotrexate, gender, age and disease duration as independent variables. Multi-collinearity effect in multivariable regression models was excluded by a stepwise approach with each variable included for *P *< 0.05 and excluded for *P *> 0.1. A tolerance test was employed to exclude models in which the sum of the values exceeded the sum of the variances for all variables. All the results are presented as two-tailed values with statistical significance if *P *values < 0.05.

As to the sample size calculation, to plan a study with a minimal pre-defined Hazard Ratio (HR) ≥1.4, an accrual interval of 3 years, and a follow-up period of 24 months, at least 222 subjects are needed to achieve a > 80% power with an 5% α error. With an expected drop-out of 25% during the follow-up, 280 subjects were enrolled in the study.

## Results

After excluding those already under treatment with TNF-α blockers (*n *= 224) and those with adequate clinical response to traditional DMARDs treatment (*n *= 545), 280 PsA subjects were enrolled in this study. Of these, 10 were missed during the follow-up (because of missing baseline data, missing follow-up visits, premature withdrawal of treatment with TNF-α blockers). Thus, 270 PsA subjects, completing their follow-up, were included in the analysis. Their clinical and demographic characteristics are reported in Table 1.

**Table 1:** Clinical and demographic characteristics of the study population

Variable	PsA subjects (number = 270)
Male gender	124 (45.9%)
Age (years)	51.73 ± 11.49
Disease subset	
Axial + peripheral	95 (35.2%)
Peripheral	95 (35.2%)
Axial	64 (23.7%)
Mutilans	16 (5.9%)
MetS	91 (33.7%)
HS	76 (28.1%)
CPs	58 (21.5%)
Concomitant MTX	111 (41.1%)
Disease duration (years)	4.87 ± 2.94
ESR (mm/h)	18.05 ± 13.19
CRP (mg/L)	2.64 ± 2.93
SJC	3.77 ± 4.08
TJC	11.93 ± 5.48
PASI	1.48 ± 0.71
HAQ	2.47 ± 1.45
VAS	76.7 ± 20.12
Patient global VAS	74.25 ± 22.38
Tender entheseal count	10.97 ± 5.40
Hypercholesterolemia	139 (51.5%)
Hypertriglyceridemia	92 (34.1%)
Impaired fasting glucose	20 (7.4%)
Hypertension	57 (21.1%)
Smoking habit	65 (24.1%)
Obesity	111 (41.1%)

CPs, presence of carotid plaques (carotid intima-media thickness > 1.3 mm); CRP, C-reactive protein; ESR, erythrocyte sedimentation rate; HAQ, Health Assessment Questionnaire; HS, hepatic steatosis; MetS, metabolic syndrome; MTX: Methotrexate; PASI, Psoriasis Area Severity Index; SJC, swollen joint count; TJC, tender joint count; VAS, Visual Analogue Scale for pain.

As to the use of TNF-α blockers, adalimumab (40 mg/every 2 weeks) was employed in 80 subjects (29.6%); etanercept (50 mg/week) in 111 (41.1%) and infliximab (5 mg/kg every 8 weeks) in 79 subjects (29.3%). A MetS was diagnosed in 84 (31.1%) subjects. The presence of CPs and of HS was found in 36 (13.3%) and 71 (26.3%) subjects, respectively. On the other hand, 163 (60.4%) PsA subjects did not have CPs or HS; 80 (29.6%) had either one of the two, and 27 (10%) had both. A direct correlation was found between CRP and ESR with the IMT values (r = 0.531, *P *< 0.001 and r = 0.476, *P *< 0.001). Whereas no difference in the prevalence of HS was found based on stratification according to different clinical subsets (Pearson's χ^2 ^for inter-groups independence *P *= 0.516), the presence of CPs was found in 10.5% of subjects with peripheral subset; 14.1% of those with axial subset, 26.3% in the axial + peripheral subset and in 87.5% of those with the mutilans subset (Pearson's χ^2 ^for inter-groups independence *P *< 0.001).

### Twelve-month follow-up (T0-T1)

During the follow-up, no changes in treatment schedules and/or in VRFs prevalence were reported in the study population. As a whole, 98/270 (36.3%) PsA individuals, achieving 5 or more of the minimal disease activity criteria [20], were classified in MDA.

MDA was achieved in 33 (41.8%) subjects receiving infliximab, in 41 (36.9%) of those receiving etanercept and in 24 (30%) of those receiving adalimumab (Pearson's χ^2 ^for inter-groups independence *P *= 0.299). As reported in Table 2 the prevalence of different clinical subsets did not differ among those who achieved MDA and those who did not. Overall, the rate of achieving MDA did not differ according to PsA clinical subsets (Pearson's χ^2 ^for inter-groups independence *P *= 0.092).

**Table 2:** Clinical and demographic characteristics of the population at baseline.

Variable	Non-MDA patients Number = 172	MDA patients Number = 98	*P*
Age (years)	52.66 ± 10.60	50.08 ± 8.81	0.042
Male gender	65 (37.8%)	59 (60.2%)	0.001
Clinical subset			
Axial + Peripheral	63 (36.6%)	32(32.7%)	0.596
Peripheral	59 (34.3%)	36 (36.7%)	0.693
Axial	41 (23.8%)	23 (23.5%)	1.000
Mutilans	9(5.2%)	7(7.1%)	0.595
MetS	73(42.4%)	18(18.4%)	< 0.001
HS	62(36.0%)	14(14.3%)	< 0.001
CPs	48(27.9%)	10(10.2%)	0.001
Disease duration (months)	101.72 ± 74.01	126.93 ± 58.83	0.004
ESR (mm/h)	14.35 ± 11.06	20.16 ± 13.86	< 0.001
CRP (mg/L)	1.66 ± 1.83	3.20 ± 3.28	< 0.001
SJC	3.75 ± 4.30	3.80 ± 3.68	0.929
TJC	12.99 ± 5.62	10.06 ± 4.71	< 0.001
PASI	1.49 ± 0.68	1.45 ± 0.76	0.700
HAQ	2.39 ± 1.41	2.61 ± 1.52	0.230
VAS	77.50 ± 17.30	75.30 ± 24.33	0.390
Patient global VAS	74.24 ± 21.08	74.28 ± 24.62	0.988
Tender entheseal count	11.71 ± 5.45	9.66 ± 5.06	0.003
Hypercholesterolemia	103(59.9%)	36(36.7%)	< 0.001
Hypertriglyceridemia	60(34.9%)	32(32.7%)	0.790
Impaired fasting glucose	11(6.4%)	9(9.2%)	0.470
Hypertension	39(22.7%)	18(18.4%)	0.441
Smoking habit	36(20.9)	29(29.6)	0.138
Obesity	88(51.2%)	23(23.5%)	< 0.001

Stratification according to the achieving of MDA at 12-month follow-up. CPs, presence of carotid plaques (carotid intima-media thickness > 1.3 mm); CRP, C-reactive protein; ESR, erythrocyte sedimentation rate; HAQ, Health Assessment Questionnaire; HS, hepatic steatosis; MetS, metabolic syndrome; PASI, Psoriasis Area Severity Index; SJC, swollen joint count; TJC, tender joint count; VAS, Visual Analogue Scale for pain.

Those who had achieved MDA were more often men (*P *= 0.001) and had a younger age (*P *= 0.042), a longer disease duration (*P *= 0.004), a higher ESR (*P *< 0.001) and CRP (*P *< 0.001), and a lower TJC (*P *< 0.001) and tender entheseal counts (*P *= 0.003) than those who did not (Table 2). Conversely, the MetS, CPs and HS (*P *always < 0.001) were more frequent in those who did not achieve MDA as compared with those who did. As to the features of the MetS, the prevalence of obesity and of hypercholesterolemia was higher in those who did not achieve MDA.

**Figure 1:**
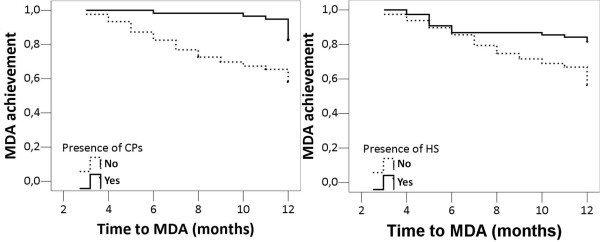
**Kaplan-Meier survival model for achieving minimal disease activity (MDA) according to the presence of CPs and HS**. CPs, presence of carotid plaques (carotid intima-media thickness > 1.3 mm); HS, hepatic steatosis; MDA, minimal disease activity.

Separate Kaplan-Meier survival models (Figure 1) showed a significant difference in those achieving MDA whether or not HS (Log-Rank: 12.894, *P *< 0.001) and CPs (Log-Rank: 12.849, *P *< 0.001) were present. After adjusting for the presence of the MetS as well as for all the other demographic characteristics and for rheumatologic and cardiovascular variables, HS and CPs independently predicted the risk of not achieving MDA (HR: 1.91, 95%CI: 1.04 to 3.38, *P *= 0.035 and HR: 3.21, 95%CI: 1.64 to 6.29, *P *= 0.001, respectively).

**Figure 2:**
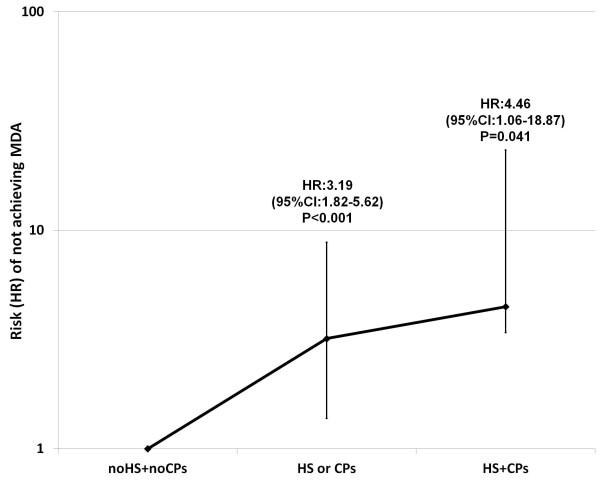
**Risk of not achieving minimal disease activity (MDA) at 12-month follow-up according to the presence of HS and/or CPs**. 95%CI, 95% confidence interval; CPs, presence of carotid plaques (carotid intima-media thickness > 1.3 mm); HR, hazard ratio; HS, hepatic steatosis; MDA, minimal disease activity.

MDA was achieved by 76 (46.6%) subjects with neither HS nor CPs, by 20 (25.0%) of those with HS or CPs and by 2 (7.4%) of those with both HS and CPs (Pearson's χ^2 ^for inter-groups independence *P *< 0.001). As compared with those with neither HS nor CPs, a progressively increasing HR of not achieving MDA was found in those with HS or CPs and in those with both HS and CPs (Figure 2).

In addition to HS and CPs, male gender (HR: 2.25, 95%CI: 1.47 to 3.43, *P *< 0.001), a low TJC (HR: 1.15, 95%CI: 1.08 to 1.23, *P *< 0.001), and a high CRP (HR: 1.18, 95%CI: 1.07 to 1.30, *P *< 0.001) independently predicted achieving MDA in this regression model.

### Twenty-four-month follow-up (T1-T2)

Among the 98 subjects who had achieved MDA during the first 12 months of follow-up, 17 (17.3%) relapsed and 81 (82.7%) maintained their MDA during the additional 12-months of follow-up (T2). In a regression model in which only the 98 subjects who had achieved MDA during the first 12-month follow-up were evaluated, the presence of HS or CPs was associated with a high risk of disease relapsing during the additional 12-months of follow-up (HR:2.85, 95%CI:1.27 to 6.37, *P *= 0.011 and HR: 3.17,95%CI:1.57 to 6.41, *P *= 0.001, respectively). Compared with subjects with neither HS nor CPs, the HR of PsA relapse was higher in those with HS or CPs (HR: 3.05, 95%CI:1.67 to 5.55, *P *< 0.001) and in those with both HS and CPs (HR: 8.93, 95%CI:1.21 to 66.66, *P *= 0.032).

## Discussion

In the setting of PsA subjects, several clinical and laboratory predictors of six-month and three-month response to TNF-α blockers have been identified [21,22]. In PsA subjects starting treatment with TNF-α blockers, this prospective study shows that HS or CPs at baseline are associated *per se *with a high risk of not achieving MDA.

A variety of variables (CRP, complement C3, IL-6 and TNF-α) implicated in the regulation of the inflammatory process [23] and, in turn, in the pathophysiology of arthritides [24], are relevant in the etiology and the development of atherosclerosis [25-27]. Major markers of atherosclerosis (HS and CPs) that assess the global damage due to metabolic and inflammatory determinants [14] are affected in PsA subjects [9,13,16]. Accordingly, subjects who exhibit a high prevalence of HS and of CPs are likely to have experienced a more severe inflammatory state [12-15].

Among the 98/270 PsA patients who achieved MDA at 12 months, the prevalence of HS and of CPs was significantly lower than in those who did not. Accordingly, the presence of one of these atherosclerosis markers was associated with a high risk of not achieving MDA. Moreover, the presence of HS or of CPs was associated with a poor probability of sustained MDA at the 24-month follow-up. However, because of the relatively small number of patients included in this sub-analysis, the evaluation of the impact of CPs and/or HS on achieving sustained MDA, although statistically significant, should be considered only as a proof of concept.

The combined data lend credence to the possibility that the presence of CPs and/or HS is a negative predictor of achieving and maintaining MDA.

By evaluating such a large number of consecutive PsA patients (270 PsA subjects), the MetS was diagnosed in 33.7%, HS was found in 28.1% and CPs in 21.5% of subjects. These data are in line with previous literature data obtained on smaller size samples [3,5,6]. Further strengthening the link between cardiometabolic variables and inflammation, in a previous analysis on the present population, we have found that obesity (defined as a Body Mass Index > 30) is associated with a statistically significant increase in the risk of a poor treatment response in PsA subjects receiving TNF-α blockers [28]. At variance with the already published data, in this study the presence of obesity is defined according to the waist circumference measurement (which is a major NCEP criterion for the diagnosis of the MetS) [19]. However, as highlighted in Table 2 also in this case, the prevalence of obesity is higher in those not achieving MDA. Moreover, since obesity is a major feature of the MetS, and being known to impact on HS and CPs presence, a multivariate regression analysis was performed to adjust all the results of this study for the presence of MetS and of its features and to exclude collinearity in the prediction of achieving MDA. In this respect, we are confident to have excluded some important bias in this study.

In addition to CPs and HS, other demographic (male gender) and rheumatologic (CRP and TJC) variables predicted the achieving of MDA. The relevance of CPs and HS as MDA predictors has been adjusted (regression model) for the large majority of clinical and laboratory variables known to predict the achieving of MDA [22].

All the carotid and liver ultrasound evaluations were performed only at the study baseline evaluation. A prospective evaluation of changes in these parameters during the treatment with TNF-α blockers could have further clarified the interrelation between IMT, HS and PsA disease activity. Although some literature data [29] suggest that treatment with 'biologic agents' is associated with the lowering of the IMT as well as of the degree of liver steatosis, further *ad hoc *designed studies are needed to address this important issue.

The link between inflammation and atherosclerosis is strongly supported by the finding of a direct correlation between CRP and ESR with the IMT in the present setting. All these inflammatory reactants have been included in the regression model to adjust for their values. The finding that the achieving of MDA is directly associated with a high CRP level and negatively associated with HS and CPs might sound like a contrasting result. However, these findings are in line with the possibility that HS and the presence of CPs are atherosclerosis markers influenced both by inflammation and by other variables (that is, obesity, aging) known to negatively impact on the achieving of MDA [22,28]. Although further studies are needed to describe better the underlying pathophysiologic mechanisms, this finding suggests that the presence of HS and CPs can be reliable markers of a cumulative (inflammatory + metabolic) damage.

A noteworthy finding is that the presence of CPs was found in 87.5% of those with the mutilans PsA subset. Because of the rarity of this clinical subset, the number of subjects evaluated was extremely low and no statistical inferences are possible from these data. However, that the CPs prevalence is maximal in the most severe PsA subset needs to be further evaluated in properly designed studies.

A series of markers is involved in the interrelation between inflammation and atherosclerosis [9,27]. In addition to ESR and CRP, the evaluation of TNF-α and IL-6 levels could have been of interest in the present setting. However, these measurements were not available in all cases.

Among PsA subjects enrolled in the present setting, the prevalence of an axial involvement is high (23.7%). At variance with most data in the literature, we have enrolled subjects experiencing a failure of treatment with traditional DMARDs and with the indication to start treatment with TNF-α blockers. Because of its high rate of refractoriness to DMARDs, the presence of an axial subset has been recognized as a major criterion to start treatment with TNF-α blockers [30]. The selection criteria used could have caused such a high prevalence of the axial subset in our study population. However (Table 2), there was no significant difference in the achieving of MDA after stratifying the population according to the different PsA clinical subsets.

Most previous studies in PsA subjects employed different criteria to define a good clinical response. We have chosen the criteria of achieving MDA proven to be appropriate in providing an outcome measure for clinical trials [21]. As to clinical and demographic variables known to predict MDA, the hazard ratios found in the present study are in line with those of previous reports [22].

In addition to their obvious pathophysiological significance, these data may have pharmaco-economic implications [31]. Because of their high cost and their side effects (that is, a higher than normal risk of infections and of malignancies) [32], the identification of predictors of the success of TNF-α blockers is mandatory. By stratifying the achieving of a good clinical response, a risk/benefit evaluation may be performed in each case and for each patient. This, in turn, would hinder the use of TNF-α blockers in subjects with a low probability of success. Further studies with higher numbers of subjects are needed to address this important issue.

## Conclusions

The presence of HS and/or CPs is a negative predictor of achieving and maintaining MDA during treatment with TNF-α blockers.

## Abbreviations

CCA: common carotid artery; CPs: carotid plaques; CRP: C-reactive protein; CV: cardiovascular; DMARDs: disease modifying anti-rheumatic drugs; ESR: erythrocyte sedimentation rate; HAQ: health assessment questionnaire; HR: hazard ratio; HS: hepatic steatosis; IFG: impaired fasting glucose; IL: interleukin; IMT: intima-media thickness; MDA: minimal disease activity; MetS: metabolic syndrome; MTX: Methotrexate; NCEP: National Cholesterol Education Program; Non-MDA: subjects not achieving MDA; PASI: psoriasis area severity index; PsA: psoriatic arthritis; SJC: swollen joint count; s-MDA: sustained MDA; TJC: tender joint count; SD: standard deviation; TNF-α: tumor necrosis factor-α; US: ultrasonography; VAS: visual analog scale for pain; VRFs: vascular risk factors.

## Competing interests

The authors declare that they have no competing interests.

## Authors' contributions

MNDDM conceived and designed the study, performed statistical analyses, interpreted results and drafted the manuscript; RP designed the study and interpreted results; SI designed the study and acquired clinical data; RL and AR acquired clinical data and drafted the manuscript; GT and RS interpreted results and performed critical revision of the manuscript. All authors read and approved the final manuscript.
